# P-512. Changes in Renal Function After Switching from Emtricitabine/Tenofovir Disoproxil Fumarate to Emtricitabine/Tenofovir Alafenamide Fumarate for HIV Pre-exposure Prophylaxis (PrEP): A Real-World Study

**DOI:** 10.1093/ofid/ofae631.711

**Published:** 2025-01-29

**Authors:** Kaori L Ito, Xiwen Huang, Seojin Park, Li Tao, Joshua Gruber

**Affiliations:** Gilead Sciences, Inc., Foster City, California; Gilead Sciences, Inc., Foster City, California; Gilead Sciences, Inc., Foster City, California; Gilead Sciences, Foster City, CA; Gilead Sciences, Foster City, CA

## Abstract

**Background:**

In the DISCOVER study (NCT02842086), emtricitabine/tenofovir alafenamide fumarate (F/TAF) showed a more favorable renal safety profile compared with emtricitabine/tenofovir disoproxil fumarate (F/TDF). However, existing evidence on renal safety of F/TAF versus F/TDF in real-world settings is limited and complicated by potential selection biases, including confounding by indication. We evaluated changes in renal function in individuals before and after switching from F/TDF to F/TAF using a crossover study design.

**Figure.**
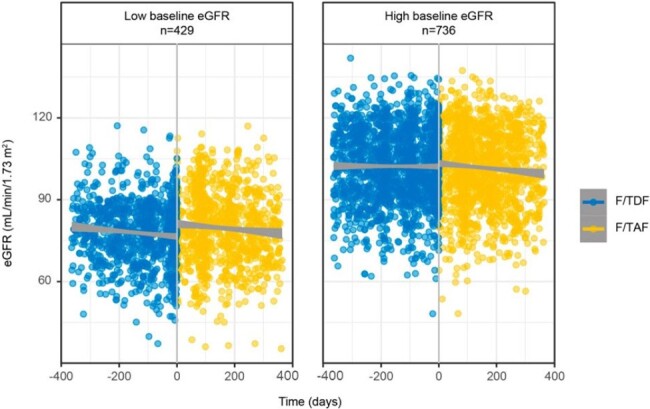
eGFR Values One Year Prior to and After Switching from F/TDF (Blue) to F/TAF (Yellow) in Individuals with Low and High Baseline eGFR* *Low and high baseline eGFR were defined as <90 mL/min/1.73 m2 and ≥90 mL/min/1.73 m2, respectively. Scatterplots with trendlines plotted separately for each treatment type. eGFR was estimated using the Chronic Kidney Disease Epidemiology Collaboration equation.

eGFR, estimated glomerular filtration rate; F/TAF, emtricitabine/tenofovir alafenamide; F/TDF, emtricitabine/tenofovir disoproxil fumarate.

**Methods:**

We conducted a retrospective, observational analysis of data from Optum Clinformatics. Eligible individuals were males who initiated F/TDF between July 2012 and May 2023, switched to F/TAF, and had ≥1 estimated glomerular filtration rate (eGFR) measurement ≤1 year pre- and post-switch. Baseline eGFR categories were defined based on the first eGFR measurement available after F/TDF initiation and categorized as low (< 90 mL/min/1.73 m^2^) and high (>90 mL/min/1.73 m^2^). Mixed-effects models were used to estimate individual-level effects, and time series analysis with segmented regression was used to compare long-term changes in eGFR pre- and post-switch across all individuals and by baseline eGFR level. Adherence to regimen was described as the proportion of days covered (PDC).

**Table.**
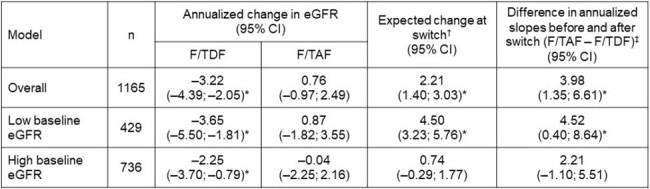
Estimated Change in Annualized eGFR after Switching from F/TDF to F/TAF for PrEP *P<0.05 for a comparison of F/TDF vs F/TAF. †Expected change in eGFR after switching from F/TDF to F/TAF was estimated using an interrupted time series model with random slopes and intercepts for each individual, calculated as: eGFR ∼ time + switch + time after switch + (time│subject). ‡Determined using a Z–test. eGFR was estimated using the Chronic Kidney Disease Epidemiology Collaboration equation. Annualized eGFR change calculated as coefficients for time (F/TDF) and time after switch (F/TAF) multiplied by 365.

eGFR, estimated glomerular filtration rate; F/TAF, emtricitabine/tenofovir alafenamide; F/TDF, emtricitabine/tenofovir disoproxil fumarate; PrEP, pre-exposure prophylaxis.

**Results:**

Among 1165 individuals, a decrease in annualized eGFR was observed while individuals were receiving F/TDF (–3.22 mL/min/1.73 m^2^; 95% CI: –4.39; –2.05), but not after switching to F/TAF (0.76; 95% CI: –0.97; 2.49); this association persisted in the low baseline eGFR subgroup but not in the high baseline eGFR subgroup (**Figure** and **Table**). The overall expected increase in eGFR immediately after switching to F/TAF was 2.21 mL/min/1.73 m^2^ (95% CI: 1.40; 3.03) and was higher for those with low baseline eGFR compared with high baseline eGFR (P< 0.001; **Table**). No significant differences were observed for mean (SD) PDC while individuals were on F/TDF (0.85 [0.22]) and on F/TAF (0.85 [0.22]).

**Conclusion:**

In this real-world analysis, individuals using PrEP experienced improvement in renal function after switching from F/TDF to F/TAF, with a greater immediate benefit among those with low baseline eGFR.

**Disclosures:**

**Kaori L. Ito, PhD, OTR/L**, Gilead Sciences, Inc.: Employee|Gilead Sciences, Inc.: Stocks/Bonds (Public Company) **Xiwen Huang, PhD**, Gilead Sciences, Inc.: Employee|Gilead Sciences, Inc.: Stocks/Bonds (Public Company) **Seojin Park, PharmD, MS**, Gilead Sciences, Inc.: Employee|Gilead Sciences, Inc.: Stocks/Bonds (Public Company) **Li Tao, MD, PhD**, Gilead Sciences, Inc.: Employee|Gilead Sciences, Inc.: Stocks/Bonds (Public Company) **Joshua Gruber, PhD MPH**, Gilead Sciences, Inc.: Employee|Gilead Sciences, Inc.: Stocks/Bonds (Public Company)

